# Triglyceride-glucose index, renal function and cardiovascular disease: a national cohort study

**DOI:** 10.1186/s12933-023-02055-4

**Published:** 2023-11-28

**Authors:** Cancan Cui, Lin Liu, Te zhang, Ling Fang, Zhanhao Mo, Yitian Qi, Jia Zheng, Zhijia Wang, Haikun Xu, Han Yan, Siqi Yue, Xuekui Wang, Zhiyuan Wu

**Affiliations:** 1 China-Japan Union Hospital of Jilin University, Jilin University, Changchun, China; 2 Centre for Precision Health, Edith Cowan University, 270 Joondalup Drive, Joondalup, WA 6027 Australia

**Keywords:** Triglyceride-glucose (TyG) index, Renal function, eGFR, Cardiovascular disease, Mediation analysis

## Abstract

**Background:**

The triglyceride-glucose (TyG) index is a predictor of cardiovascular diseases; however, to what extent the TyG index is associated with cardiovascular diseases through renal function is unclear. This study aimed to evaluate the complex association of the TyG index and renal function with cardiovascular diseases using a cohort design.

**Methods:**

This study included participants from the China Health and Retirement Longitudinal Study (CHARLS) free of cardiovascular diseases at baseline. We performed adjusted regression analyses and mediation analyses using Cox models. The TyG index was calculated as Ln [fasting triglyceride (mg/dL) × fasting glucose (mg/dL)/2]. Renal function was defined by the estimated glomerular filtration rate (eGFR).

**Results:**

A total of 6 496 participants were included in this study. The mean age of the participants was 59.6 ± 9.5 years, and 2996 (46.1%) were females. During a maximum follow-up of 7.0 years, 1 996 (30.7%) people developed cardiovascular diseases, including 1 541 (23.7%) cases of heart diseases and 651 (10.0%) cases of stroke. Both the TyG index and eGFR level were significantly associated with cardiovascular diseases. Compared with people with a lower TyG index (median level) and eGFR ≥ 60 ml/minute/1.73 m^2^, those with a higher TyG index and decreased eGFR had the highest risk of cardiovascular diseases (HR, 1.870; 95% CI 1.131–3.069). Decreased eGFR significantly mediated 29.6% of the associations between the TyG index and cardiovascular diseases.

**Conclusions:**

The combination of a higher TyG index and lower eGFR level was associated with the highest risk of cardiovascular diseases. Renal function could mediate the association between the TyG index and cardiovascular risk.

**Supplementary Information:**

The online version contains supplementary material available at 10.1186/s12933-023-02055-4.

## Background

Insulin resistance has been identified as an independent risk factor for the onset of cardiovascular diseases [1], which has caused an enormous burden given the high morbidity and mortality rates [2]. The triglyceride-glucose (TyG) index has been proposed as a reliable indicator of insulin resistance [3, 4], which also plays an important role in cardiovascular diseases [5]. Recently, a considerable number of studies have provided strong evidence suggesting that the TyG index is associated with the development and prognosis of cardiovascular diseases, including stable coronary artery disease, carotid plaque, coronary artery calcification, and acute coronary syndrome [6–8]. In addition, the TyG index is also closely associated with risk factors for cardiovascular diseases, such as arterial stiffness and hypertension [9, 10]. Thus, the identification of insulin resistance markers is essential for the early prevention of cardiovascular diseases.

Renal function is another important factor in cardiovascular diseases [11]. Studies have shown that multiple biomarkers of renal function are closely associated with the development and prognosis of cardiovascular diseases, including estimated glomerular filtration rate (eGFR) [12]. Insulin resistance is a systemic disorder that affects many organs and insulin-regulated pathways [13]. Insulin also influences the kidney because the insulin receptor is expressed on renal tubular cells and podocytes [14]. The strong correlation of salt-sensitive arterial hypertension with insulin resistance indicates the involvement of the kidney in insulin resistance and cardiovascular diseases [15]. A recent study also pointed out that the impact of insulin resistance on mortality in individuals with albuminuric diabetic kidney disease may be mediated by its relationship with albuminuria [16]. However, the complex association between the TyG index, renal function and cardiovascular diseases needs more evidence for a better understanding of the TyG index as an important risk factor for cardiovascular diseases.

Therefore, this study aimed to investigate the combined association between the TyG index and eGFR level with the onset of cardiovascular diseases given that insulin resistance and renal function are two initial predictors of cardiovascular health. In addition, we tested the hypothesis that the association between the TyG index and cardiovascular diseases is partially mediated by impaired renal function.

## Methods

### Study population

This current study was a secondary analysis of the China Health and Retirement Longitudinal Study (CHARLS), which is a national population-based cohort study (http://charls.pku.edu.cn/) among Chinese adults aged 45 years or older with four regular biannual surveys between 2011 and 2018. The participants were recruited from both rural and urban areas using a multistage stratified probability proportional-to-size sampling strategy and covered 150 counties or districts of 28 provinces in China. Details of the study design and cohort profile have been previously described [17]. The CHARLS study was performed in accordance with the principles of the Declaration of Helsinki and was approved by the Institutional Review Board of Peking University (IRB00001052-11015). All participants provided written informed consent before participating in the CHARLS study.

**Fig. 1:**
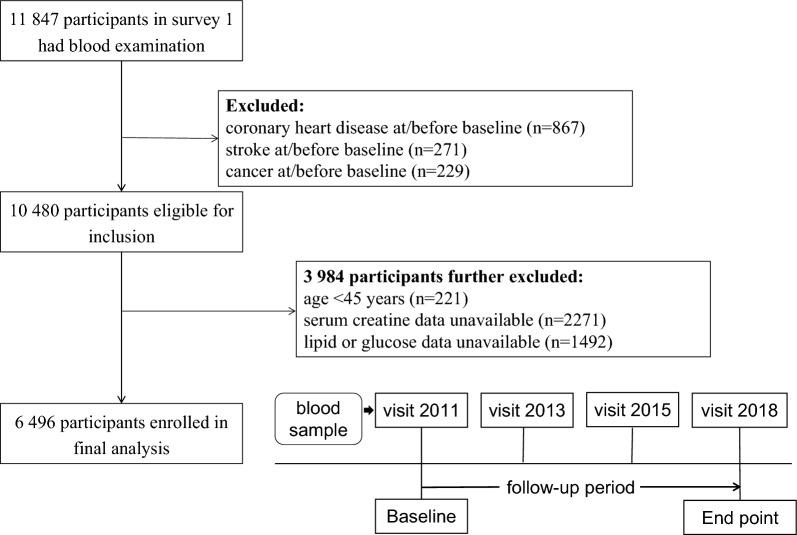
Flowchart and follow-up setting of this current study

At each survey, the trained staff conducted face-to-face interviews to collect the sociodemographic characteristics, medical history, health behavior, cognitive function, and depressive status using a standardized questionnaire [18]. In this current study, participants who underwent the visit between 2011 and 2012 were included as baseline and then followed at the three subsequent surveys. Those lacking blood sample tests or with cardiovascular diseases or cancer history at baseline were excluded. Finally, a total of 6 496 participants were included in the final analysis. The detailed inclusion and exclusion process is shown in Fig. 1. This study was conducted following the Strengthening the Reporting of Observational Studies in Epidemiology (STROBE) reporting guidelines.

### Exposure and outcome

Fasting venous blood samples were collected by medical staff from the Chinese Centre for Disease Control and Prevention based on the standard protocol and subsequently tested at the central laboratory. Triglycerides and glucose were measured based on an enzymatic colorimetric test. The coefficient of variation of triglycerides was 1.5% within the assay. The coefficient of variation of glucose was 0.9% within the assay. Non-high-density lipoprotein (non-HDL) cholesterol was calculated as total cholesterol minus HDL cholesterol. Serum creatinine (µmol/L) was measured by the rate-blanked and compensated Jaffe creatinine method. High-sensitivity C-reactive protein (hs-CRP) was measured based on an immunoturbidimetric assay on a Hitachi 7180 chemistry analyzer (Hitachi, Tokyo, Japan). The coefficient of variation (CV) of blood marker measurement was < 5%.

According to previous studies, the TyG index was calculated as Ln [triglycerides (mg/dL) × glucose (mg/dL)/2]. The eGFR level (mL/min/1.73 m^2^) was calculated according to the 2021 Chronic Kidney Disease Epidemiology Collaboration (CKD-EPI) [19]. Impaired renal function was defined as an eGFR < 60 mL/min/1.73 m^2^.

The study outcome was any incidence of cardiovascular diseases of coronary heart disease (including heart attack and coronary heart disease) and stroke during the follow-up period (from wave 2 to wave 4). Information on cardiovascular diagnosis history was collected using the standardized question: ‘Have you been told by a doctor that you have been diagnosed with a heart attack, coronary heart disease or stroke?’. The outcomes were assessed by rigorously trained interviewers through standardized questionnaires that are harmonized to international leading aging surveys in the Health and Retirement Study (HRS) and related international aging surveys, including the English Longitudinal Study of Aging (ELSA) and the Survey of Health, Aging and Retirement in Europe (SHARE). Quality control of data recording and checking was conducted to ensure data reliability. Participants who reported heart disease or stroke during the follow-up period were defined as having incident cardiovascular diseases that have been validated and widely used [20].

### Covariates

Baseline measurements of age, sex, education level, marital status, residence location, BMI, smoking, and self-reported health conditions (hypertension and diabetes) were included as covariates in the current study. Educational level was categorized as “primary education,” “secondary education,” and “third education.” Marital status included “married” and “others.” Residence location included “urban” and “rural.” Smoking status was defined as “never smoking”, “current smoker” and “former smoker”. BMI was calculated as weight (in kilograms)/height^^2^ (in meters squared) and grouped into normal weight (BMI < 24.0 kg/m^2^), overweight (BMI ≥ 24.0 kg/m^2^) and obesity (BMI ≥ 28.0 kg/m^2^) according to the overweight and obesity standard for the Asian population [21]. Hypertension was defined as systolic blood pressure ≥ 140 mmHg or diastolic blood pressure ≥ 90 mmHg or self-reported diagnosis history of hypertension or use of any antihypertensive medication [22]. Diabetes was defined as fasting glucose ≥ 7.0 mmol/L or self-reported diagnosis history of diabetes or use of any hypoglycemic medication [23]. The definition of metabolic syndrome is defined according to the National Cholesterol Education Program (NCEP) Adult Therapy Group III (ATP III) as the presence of three or more of the following five criteria: waist circumference over 90 cm (men) or 80 cm (women), which is adapted to the Asian population; blood pressure over 130/85 mmHg or self-reported history of hypertension or current use of antihypertensive medication; fasting triglyceride level over 150 mg/dl or current use of lipid-lowering medication; fasting high-density lipoprotein cholesterol level less than 40 mg/dl (men) or 50 mg/dl (women); and fasting blood sugar over 100 mg/dl or self-reported history of diabetes or current use of antidiabetic medication following a previous study [24].

### Statistical analysis

Baseline characteristics were described using the mean (standard deviation, SD) for continuous variables and frequency (proportion) for categorical variables. To determine the association between the TyG index, impaired renal function, and the development of cardiovascular diseases, multivariable-adjusted Cox regression models were used to calculate the hazard ratio (HR) with 95% confidence interval (CI), considering the time-to-event framework. Age (continuous), sex, residence (rural, urban), education level (primary, secondary, third), marital status (married, others), smoking status (current, former, never), BMI (continuous), hypertension (yes, no), diabetes (yes, no), and nonHDL cholesterol (continuous) were included in the adjusted model. The TyG index was grouped into two groups according to the median value. Then, participants were grouped into four categories according to the joint assessment of the TyG index and eGFR value. In addition, the participants were grouped into nine categories according to the joint assessment of the TyG index (using tertile) and eGFR value (60 and 90 ml/minute/1.73 m^2^ as cutoff points). The dose–response relationship of the TyG index and eGFR level with cardiovascular risk was shown using the restricted cubic spline function using 3 knots at the 10th, 50th, and 90th percentiles. The reference point was set as the median value of varibales among the corresponding populations. We performed multiple sensitivity analyses after additionally adjusting for hs-CRP level (continuous), using 130/80 mmHg to define hypertension, and repeating the analyses using multiple imputed analyses (5 iterations) by Markov chain Monte Carlo. In addition, we reanalyzed the effect of the TyG index and eGFR level on cardiovascular risk using propensity scores of the inverse probability treatment weighting (IPTW) method. The sample weights in the cohort survey procedures were also considered using weighted regression. Furthermore, we performed subgroup analysis under two scenarios. First, we repeated the analysis among those without treatment for diabetes, hypertension or dyslipidemia at baseline. Then, we further excluded those with treatment for diabetes, hypertension or dyslipidemia during follow-up. We also performed analysis stratified by obesity, diabetes, hypertension and metabolic syndrome.

We conducted a mediation analysis to assess the direct and indirect associations between the TyG index group and cardiovascular diseases via impaired renal function. In brief, the TyG index group (< median value of 8.6) was used as a predictor variable (X), decreased eGFR (< 60 mL/min/1.73 m^2^) as a mediator (M) and cardiovascular disease onset as the outcome variable (Y). The analysis included four steps: (1) establishing that X is associated with Y (Model Y = βTot X) (βTot = total effect); (2) establishing that X is associated with M (Model M = β1 X) (β1 = indirect effect1); (3) determining which part of Y is explained by controlling for X (Model Y = β2 M + βDir X) (β2 = indirect effect, βDir = direct effect); and (4) calculating the proportion of indirect or mediation effect: mediation effect (%) = (β1 × β2/βTol) × 100%. This method has been widely used in previous studies to quantify the mediating effect [25].

All statistical analyses were performed using R software (version 4.2.1). Weighted regression was performed using the ‘survey’ package. Mediation analysis was performed using the ‘mediation’ package, and the IPTW procedure was performed using the ‘PSweight’ package. A two-sided P value < 0.05 was considered statistically significant.

## Results

### Characteristics

A total of 6 496 participants in CHARLS from 2011 to 2018 were included in the final analyses. The mean (SD) age was 59.6 (9.5) years, including 2996 (46.1%) females (Table 1). During a maximum follow-up of 7.0 years, 1 996 (30.7) people developed cardiovascular diseases, including 1 541 (23.7%) cases of heart diseases and 651 (10.0%) cases of stroke. There were 1 194 (22.3%) participants with obesity, 2770 (42.6%) with hypertension and 1197 (18.4%) with diabetes.

**Table 1:** Characteristics of 6 496 participants

Characteristics	
Participants, No	6496
Age, years, mean (SD)	59.57 (9.53)
Sex, Female, n (%)	2996 (46.1)
Residence, n (%)	
Rural	5225 (80.5)
Urban	1267 (19.5)
Marriage, married, n (%)	5727 (88.2)
Educational level, n (%)	
Primary	4455 (68.6)
Secondary	1302 (20.1)
Third	734 (11.3)
Smoking status, n (%)	
Never	3892 (60.0)
Former	456 (7.0)
Current	2139 (33.0)
BMI^a^, kg/m^2^	23.76 (6.04)
< 23.9	3025 (56.5)
24–27.9	1137 (21.2)
≥ 28	1194 (22.3)
SBP, mmHg, mean (SD)	131.13 (21.98)
Hypertension, n (%)	2770 (42.6)
Diabetes, n (%)	1197 (18.4)
Glucose, mg/dL, mean (SD)	109.60 (34.75)
Triglycerides, mg/dL, mean (SD)	132.00 (92.19)
NonHDL cholesterol, mg/dL, mean (SD)	142.40 (38.25)

Data are presented as the mean (SD) or number (%), as appropriate

*SD* standard deviation, *BMI* body mass index, *SBP* systolic blood pressure, *HDL* high-density lipoprotein

^a^Calculated as weight in kilograms divided by height in meters squared

### TyG index, eGFR and cardiovascular diseases

**Fig. 2:**
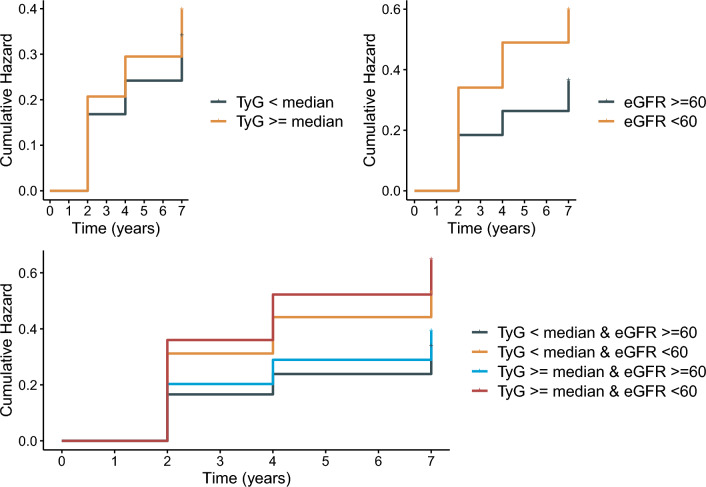
K-M plot of cardiovascular diseases by TyG index and eGFR level

**Fig. 3:**
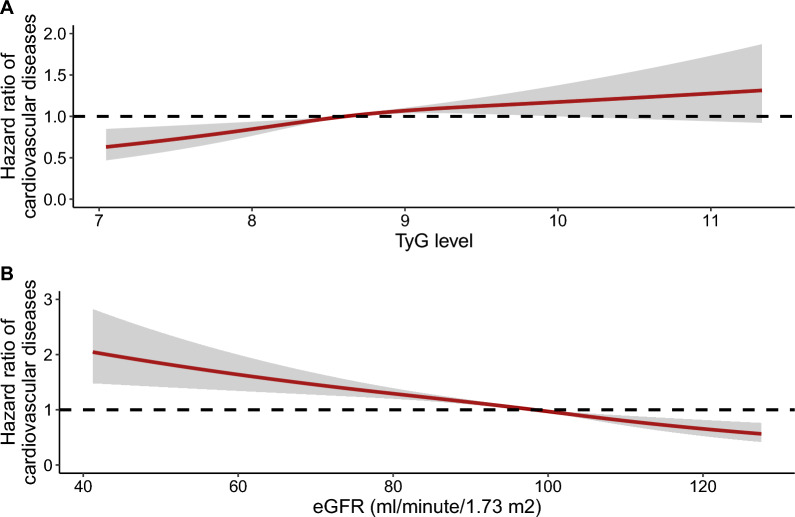
Dose-responsive relationship of the TyG index and eGFR level with the risk of cardiovascular diseases

Figure 2 shows the cumulative incidence rates of cardiovascular diseases when jointly assessing the baseline TyG index and eGFR value, and the highest rate of cardiovascular diseases was observed among those with a higher TyG index and impaired renal function. There was a positive relationship and a negative relationship of the TyG index and eGFR with cardiovascular diseases when analyzed as continuous variables (Fig. 3).

In the fully adjusted model, when compared with people with a lower TyG index (less than median level) and eGFR ≥ 60 ml/minute/1.73 m^2^, those with a higher TyG index and decreased eGFR had the highest risk of cardiovascular diseases (HR, 1.870; 95% CI 1.131–3.069), followed by those with a lower TyG index and decreased eGFR level and those with a higher TyG and normal eGFR level, as shown in Table 2.

**Table 2:** Associations of the TyG index and eGFR with the risk of cardiovascular diseases

	Unadjusted		Adjusted	
	HR (95% CI)	P value	HR (95% CI)	P value
TyG < median	Ref			
TyG ≥ median	1.183 (1.064–1.316)	0.002	1.131 (1.020–1.279)	0.041
eGFR ≥ 60	Ref			
eGFR < 60	1.799 (1.287–2.506)	0.001	1.606 (1.092–2.347)	0.015
TyG < median & eGFR ≥ 60	Ref			
TyG < median & eGFR < 60	1.644 (0.954–2.782)	0.067	1.461 (0.789–2.634)	0.215
TyG ≥ median & eGFR ≥ 60	1.173 (1.054–1.307)	0.004	1.121 (0.991–1.268)	0.071
TyG ≥ median & eGFR < 60	2.183 (1.418–3.351)	< 0.001	1.870 (1.131–3.069)	0.014

eGFR was calculated using the Chronic Kidney Disease Epidemiology Collaboration (CKD-EPI) equation; unit of eGFR: ml/minute/1.73 m^2^; median value of the TyG index: 8.6

Age, sex, residence, marriage, education level, BMI level, smoking status, hypertension, diabetes, and nonHDL cholesterol were adjusted

*HR* hazard ratio, *CI*, confidence interval, *eGFR* estimated glomerular filtration rate, *TyG* triglyceride-glucose index, *HDL* high density lipoprotein

**Fig. 4:**
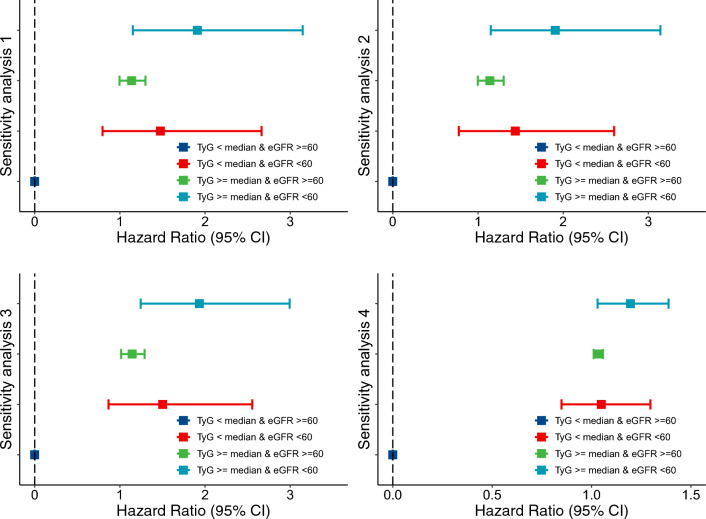
Sensitivity analyses of the combination assessment of the TyG index and eGFR level with the risk of cardiovascular diseases. Age, sex, residence, marriage, education level, BMI, smoking status, hypertension, diabetes, and nonHDL cholesterol were adjusted. Sensitivity analyses 1: additionally adjusted for hs-CRP level; sensitivity analyses 2: using 130/80 mmHg to define hypertension; sensitivity analyses 3: repeating the analyses using multiple imputed analyses (5 iterations) by Markov chain Monte Carlo; sensitivity analyses 4: using propensity scores of inverse probability treatment weighting (IPTW) method

The results remained consistent in multiple sensitivity analyses, as summarized in Fig. 4. The highest RR values were largely similar when additionally adjusting for systolic blood pressure and hs-CRP and using imputed data sets, except that the risk was significant but attenuated after IPTW weighting. In the weighted regression analysis, the results were almost consistent, although the effect size was relatively attenuated (Additional file 1: Table S2). When stratifying TyG by tertile and eGFR by 60 and 90 ml/minute/1.73 m^2^, the highest HR was still observed among those with the highest TyG and eGFR less than 60 ml/minute/1.73 m^2^ (Additional file 1: Table S3). In the subgroup analysis, there were 1060 participants treated for diabetes, hypertension or dyslipidemia at baseline. The highest HR values were still observed among those with high TyG levels and decreased eGFR. In addition, 2857 participants initiated treatment for diabetes, hypertension or dyslipidemia during follow-up. After excluding those with any treatment for diabetes, hypertension or dyslipidemia during follow-up, the results were almost consistent, as shown in Table 3. Table 4 shows the association between the TyG index and eGFR level with cardiovascular diseases stratified by obesity, diabetes, hypertension and metabolic disease.

**Table 3:** Subgroup analysis for the effect of the TyG index and eGFR on the risk of cardiovascular diseases

	Subgroup 1		Subgroup 2	
	HR (95% CI)	P value	HR (95% CI)	P value
TyG < median & eGFR ≥ 60	Ref			
TyG < median & eGFR < 60	1.462 (0.783–2.648)	0.218	1.484 (0.795–2.690)	0.201
TyG ≥ median & eGFR ≥ 60	1.183 (1.052–1.330)	0.005	1.162 (1.033–1.307)	0.013
TyG ≥ median & eGFR < 60	1.928 (1.215–3.032)	0.005	1.891 (1.191–2.976)	0.006

eGFR was calculated using the Chronic Kidney Disease Epidemiology Collaboration (CKD-EPI) equation; unit of eGFR: ml/minute/1.73 m^2^; median value of the TyG index: 8.6

Age, sex, residence, marriage, education level, BMI level, smoking status, hypertension, diabetes, and nonHDL cholesterol were adjusted

Subgroup 1: among 5436 participants without treatments for diabetes, hypertension or dyslipidemia at baseline; subgroup 2: among 3019 participants without treatments for diabetes, hypertension or dyslipidemia at baseline or during follow-up

*RR* risk ratio, *CI* confidence interval, *eGFR* estimated glomerular filtration rate, *TyG* triglyceride-glucose index, *HDL* high density lipoprotein

**Table 4:** Association of the TyG index and eGFR with the risk of cardiovascular diseases stratified by obesity, diabetes, hypertension and metabolic syndrome

	HR (95% CI)	P value
TyG < median & eGFR ≥ 60	Ref	
Non obesity		
TyG < median & eGFR < 60	1.301 (0.668–2.470)	0.426
TyG ≥ median & eGFR ≥ 60	1.122 (0.979–1.285)	0.098
TyG ≥ median & eGFR < 60	2.251 (1.257–4.048)	0.006
Obesity		
TyG < median & eGFR < 60	0.857 (0.121–4.085)	0.856
TyG ≥ median & eGFR ≥ 60	0.687 (0.215–1.887)	0.491
TyG ≥ median & eGFR < 60	1.296 (1.006–1.671)	0.045
Non diabetes		
TyG < median & eGFR < 60	1.089 (0.607–1.905)	0.768
TyG ≥ median & eGFR ≥ 60	1.129 (0.991–1.274)	0.05
TyG ≥ median & eGFR < 60	1.871 (1.061–3.289)	0.029
Diabetes		
TyG < median & eGFR < 60	1.564 (0.693–3.971)	0.141
TyG ≥ median & eGFR ≥ 60	1.225 (0.934–1.685)	0.138
TyG ≥ median & eGFR < 60	2.628 (1.784–3.338)	0.045
Non hypertension		
TyG < median & eGFR < 60	1.468 (0.706–2.991)	0.293
TyG ≥ median & eGFR ≥ 60	1.124 (0.975–1.296)	0.107
TyG ≥ median & eGFR < 60	1.480 (1.071–2.806)	0.031
Hypertension		
TyG < median & eGFR < 60	0.996 (0.415–2.241)	0.993
TyG ≥ median & eGFR ≥ 60	1.239 (1.048–1.464)	0.012
TyG ≥ median & eGFR < 60	2.079 (1.145–3.765)	0.015
Non metabolic syndrome		
TyG < median & eGFR < 60	1.254 (0.578–2.624)	0.554
TyG ≥ median & eGFR ≥ 60	1.044 (0.891–1.222)	0.597
TyG ≥ median & eGFR < 60	2.387 (1.581–3.214)	0.048
Metabolic syndrome		
TyG < median & eGFR < 60	1.214 (0.541–2.623)	0.626
TyG ≥ median & eGFR ≥ 60	1.295 (1.114–1.508)	0.001
TyG ≥ median & eGFR < 60	1.992 (1.191–3.323)	0.008

Age, sex, residence, marriage, education level, BMI level, smoking status, hypertension, diabetes, and nonHDL cholesterol were adjusted, if not stratified

**Fig. 5:**
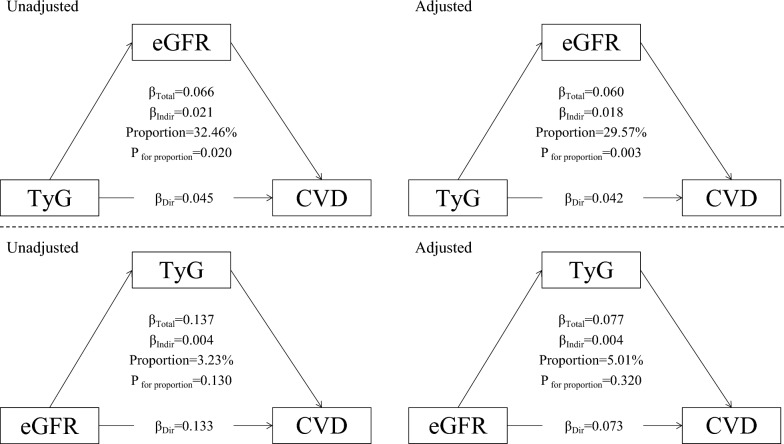
Mediation effect of renal function between the TyG index and cardiovascular diseases

Figure 5 summarizes the potential mediating effect of decreased eGFR between the TyG index and cardiovascular diseases. The mediation proportions of decreased eGFR were 32.5% (P = 0.045) and 29.6% (P = 0.042) in the unadjusted and adjusted analyses, respectively. However, we did not observe a significant mediation effect of TyG between decreased renal function and cardiovascular diseases.

## Discussion

Among 6 496 Chinese adults aged 45 years or above followed up to 7.0 years, a higher baseline TyG index and decreased eGFR were significantly associated with a higher risk of cardiovascular diseases. The highest risk of cardiovascular disease onset was observed among those with a higher TyG index and decreased eGFR. The associations persisted even after adjustment for other established cardiovascular risk factors and IPTW procedures. In addition, this study indicated that decreased eGFR partially mediated the relationship between the TyG index and cardiovascular diseases.

The positive association between the TyG index and cardiovascular diseases has been widely evaluated in previous studies. The Tehran Lipid and Glucose Study among 7521 Iranian males found that the TyG index is significantly associated with an increased risk of cardiovascular diseases or coronary heart diseases [26]. The Kailuan study reported that elevated levels of the baseline and long-term TyG index are associated with an increased risk of myocardial infarction [27]. Using National Health Insurance Service data, a Korean study found that a higher TyG index precedes and significantly predicts future ischemic heart disease among the nondiabetic population [28]. The Atherosclerosis Risk in Communities (ARIC) Study showed that a higher TyG index is independently associated with an increased risk of incident peripheral arterial disease [29]. Several studies have also reported that the TyG index, as a simple and low-cost marker, could predict the development of cardiovascular diseases [30, 31]. In addition, the cumulative exposure, variability, and progressive trajectory of the TyG index have also been linked with a higher risk of cardiovascular diseases [29, 32–34].

In addition, the association between the TyG index and other established risk factors for cardiovascular diseases has been evaluated among several cohort designs. The Hanzhong Adolescent Hypertension Cohort study found that elevated levels of the baseline TyG index and a higher long-term trajectory of the TyG index were independently associated with increased arterial stiffness [35], which was also observed in one of our previous studies [9]. In addition, the effects of TyG on diabetes, carotid atherosclerosis, hypertension, liver diseases and frailty have also been reported [36–40]. Of note, the TyG index is potentially associated with impaired renal function. The Tehran Lipid and Glucose Study recently reported that an increasing trend of homeostatic model assessment of insulin resistance (HOMA-IR) is associated with a higher risk of chronic kidney diseases [41]. Among clinical insulin resistance surrogate markers, the TyG index is significantly associated with a higher risk of reduced renal function [41]. Another study also indicated that an elevated TyG index was significantly associated with a higher risk of nephric microvascular damage [42]. The association between the TyG index and renal function also exists among specific populations, such as diabetes patients [43] and acute coronary syndrome patients [44]. Given that renal function has been validated as a prominent risk factor for cardiovascular events and the close correlation between the TyG index and renal function, we hypothesized that renal function could mediate the effect of the TyG index on cardiovascular diseases. We found that reduced renal function potentially mediates approximately 30% of the association between the TyG index and cardiovascular diseases. In addition, the combined assessment of the TyG index and eGFR could inform and stratify those with the highest cardiovascular risk.

There are possible mechanisms underlining the complex correlation among the TyG index or insulin resistance, renal function, and cardiovascular damage. Generally, insulin resistance plays an important role in metabolic disease-induced chronic kidney disease by causing hyperglycemia and later low-grade inflammation and fibrosis [13]. Insulin causes vasodilation by enhancing endothelial nitric oxide production through activation of the phosphatidylinositol 3-kinase pathway. Under insulin-resistant states, the mitogen-activated protein kinase pathway stimulates vasoconstriction and causes kidney vascular damage.

Several limitations of the current study should be acknowledged. First, owing to the observational nature of the study, we could not confirm the causal association among the TyG index, renal function and cardiovascular risk. Second, although potential cardiac risk factors were adjusted for, we still cannot exclude the possibility of residual or unmeasured confounding given the observational study design of the present analysis. Additionally, HbA1c data were not available in this current analysis, which could affect the definition of diabetes. Clinical information on proteinuria was not available, which could cause bias in the assessment of renal function. Third, the diagnosis of cardiovascular events was self-reported. Medical records were not available in the CHARLS; however, some other large-scale studies, such as the English Longitudinal Study of Aging, found that self-reported incident cardiovascular disease had good agreement with medical records. Fourth, the CHARLS focuses on general Chinese participants aged 45 years and older, and the association found in this study may not be fully generalizable to the general population or disease population. Further studies are needed for validation in other populations and health conditions.

## Conclusions

In a prospective cohort of Chinese adults, we found that reduced renal function significantly mediated the association between insulin resistance, that is the TyG index, and cardiovascular diseases. The findings recommend the combined assessment of the TyG index and renal function markers to further stratify the risk of cardiovascular diseases. Future research is needed to validate the clinical effect of targeting insulin resistance on renal function and cardiovascular health.

### Supplementary Information

**Additional file 1: Table S1.** Characteristics of 6 496 participants categorized by TyG and eGFR levels. **Table S2.** Associations of TyG index and eGFR with risk of cardiovascular diseases using sample weighted method. **Table S3.** Effect of TyG index and eGFR with risk of cardiovascular diseases when analyzed using 3*3 matrix.

## Data Availability

The datasets used and/or analyzed during the current study are publicly available or from the corresponding author upon reasonable request.

## Abbreviations

CHARLS: China health and retirement longitudinal study
TyG: Triglyceride-glucose index
hs-CRP: High-sensitivity C-reactive protein
HDL: High-density lipoprotein
BMI: Body mass index
IPTW: Inverse probability treatment weighting
CKD-EPI: Chronic kidney disease epidemiology collaboration
eGFR: Estimated glomerular filtration rate
