# Supplementary material for: Triglyceride-glucose index, renal function and cardiovascular disease: a national cohort study
Source: Cardiovasc Diabetol. 2023 Nov 28;22:325. doi: 10.1186/s12933-023-02055-4 (PMC10685637; doi:10.1186/s12933-023-02055-4)
Supplement: Supplementary file 1 — Additional file 1: Table S1. Characteristics of 6 496 participants categorized by TyG and eGFR levels. Table S2. Associations of TyG index and eGFR with risk of cardiovascular diseases using sample weighted method. Table S3. Effect of TyG index and eGFR with risk of cardiovascular diseases when analyzed using 3*3 matrix. [file 12933_2023_2055_MOESM1_ESM.docx]

**Additional file Tables**

Table S1: Characteristics of 6 496 participants categorized by TyG and eGFR levels

|  | TyG<median&eGFR≥60 | TyG<median&eGFR<60 | TyG≥median&eGFR≥60 | TyG≥median&eGFR<60 |
| --- | --- | --- | --- | --- |
| Age, years, mean (SD) | 59.29(9.57) | 72.93(9.70) | 59.33(9.17) | 69.54(9.02) |
| Sex, Female, n (%) | 1635(51.3) | 29(50.0) | 1298(41.1) | 34(39.1) |
| Residence, n (%) |  |  |  |  |
| Rural | 2655(83.3) | 44(75.9) | 2468(78.2) | 58(66.7) |
| Urban | 534(16.7) | 14(24.1) | 690(21.8) | 29(33.3) |
| Marriage, married, n (%) | 2820(88.4) | 41(70.7) | 2804(88.7) | 62(71.3) |
| Educational level, n (%) |  |  |  |  |
| Primary | 2177(68.2) | 48(82.8) | 2163(68.5) | 67(77.0) |
| Secondary | 654(20.5) | 6(10.3) | 632(20.0) | 10(11.5) |
| Third | 359(11.3) | 4(6.9) | 361(11.4) | 10(11.5) |
| Smoking status, n (%) |  |  |  |  |
| Never | 1882(59.1) | 39(67.2) | 1917(60.7) | 54(62.1) |
| Former | 224(7.0) | 1(1.7) | 224(7.1) | 7(8.0) |
| Current | 1079(33.9) | 18(31.0) | 1016(32.2) | 26(29.9) |
| BMI a, kg/m2 |  |  |  |  |
| <23.9 | 1468(55.8) | 34(72.3) | 1487(56.9) | 36(54.5) |
| 24-27.9 | 559(21.2) | 6(12.8) | 560(21.4) | 12(18.2) |
| ≥28 | 604(23.0) | 7(14.9) | 565(21.6) | 18(27.3) |
| SBP, mmHg, mean (SD) | 130.98(22.04) | 133.78(25.68) | 131.14(21.76) | 134.93(24.63) |
| Hypertension, n (%) | 1364(42.8) | 26(44.8) | 1333(42.2) | 47(54.0) |
| Diabetes, n (%) | 280(8.8) | 4(6.9) | 877(27.7) | 36(41.4) |
| Glucose, mg/dL, mean (SD) | 97.93(14.59) | 100.12(13.78) | 120.92(43.68) | 132.76(51.92) |
| Triglycerides, mg/dL, mean (SD) | 77.06(21.34) | 78.09(22.52) | 186.37(102.01) | 207.17(118.32) |
| nonHDL cholesterol, mg/dL, mean (SD) | 127.12(31.28) | 127.56(30.45) | 157.52(38.49) | 162.99(38.89) |

Data are presented as mean (SD) or number (%), as appropriate.

Abbreviation: SD, standard deviation; BMI, body mass index; SBP, systolic blood pressure; HDL, high density lipoprotein.

^a^ Calculated as weight in kilograms divided by height in meters squared.

Table S2: Associations of TyG index and eGFR with risk of cardiovascular diseases using sample weighted method

|  | Unadjusted | | Adjusted | |
| --- | --- | --- | --- | --- |
|  | HR (95% CI) | P value | HR (95% CI) | P value |
| TyG < median & eGFR ≥60 | Ref |  |  |  |
| TyG < median & eGFR <60 | 1.564(0.847-2.810) | 0.141 | 1.181(0.634-2.142) | 0.591 |
| TyG ≥ median & eGFR ≥60 | 1.159(1.029-1.306) | 0.015 | 1.139(0.997-1.302) | 0.056 |
| TyG ≥ median & eGFR <60 | 2.076(1.262-3.394) | 0.004 | 1.591(1.054-2.631) | 0.042 |

Abbreviation: HR, hazard ratio; CI, confidence interval; eGFR, estimated glomerular filtration rate; TyG, triglyceride-glucose index; HDL, high density lipoprotein

eGFR was calculated using CKD-EPI (Chronic Kidney Disease Epidemiology Collaboration) equation; unit of eGFR: ml/minute/1.73 m^2^; median value of TyG index: 8.6

Age, sex, residence, marriage, education level, BMI group, smoking status, hypertension, diabetes, and nonHDL cholesterol were adjusted.

**Table S3**: Effect of TyG index and eGFR with risk of cardiovascular diseases when analyzed using 3*3 matrix.

|  | HR (95% CI) | P value |
| --- | --- | --- |
| TyG tertile 1 & eGFR ≥90 | Ref |  |
| TyG tertile 1 & eGFR 60-90 | 1.437(1.155-1.783) | 0.001 |
| TyG tertile 1 & eGFR <60 | 1.737(0.904-3.236) | 0.087 |
| TyG tertile 2 & eGFR ≥90 | 1.114(0.953-1.302) | 0.176 |
| TyG tertile 2 & eGFR 60-90 | 1.651(1.347-2.021) | <0.001 |
| TyG tertile 2 & eGFR <60 | 1.975(0.969-3.918) | 0.054 |
| TyG tertile 3 & eGFR ≥90 | 1.288(1.102-1.507) | 0.002 |
| TyG tertile 3 & eGFR 60-90 | 1.583(1.303-1.921) | <0.001 |
| TyG tertile 3 & eGFR <60 | 2.741(1.684-4.458) | <0.001 |

Abbreviation: HR, hazard ratio; CI, confidence interval; eGFR, estimated glomerular filtration rate; TyG, triglyceride-glucose index; HDL, high density lipoprotein

eGFR was calculated using CKD-EPI (Chronic Kidney Disease Epidemiology Collaboration) equation; unit of eGFR: ml/minute/1.73 m^2^; TyG was grouped by tertile; eGFR was grouped by 60 and 90 ml/minute/1.73 m^2^

Age, sex, residence, marriage, education level, BMI group, smoking status, hypertension, diabetes, and nonHDL cholesterol were adjusted
